# Commenting on the “Great Debate”: General Abilities, Specific Abilities, and the Tools of the Trade

**DOI:** 10.3390/jintelligence7010005

**Published:** 2019-02-18

**Authors:** Margaret E. Beier, Harrison J. Kell, Jonas W. B. Lang

**Affiliations:** 1 Department of Psychological Sciences, Rice University, Houston, TX 77005, USA; 2 Academic to Career Research Center, Research & Development, Educational Testing Service, Princeton, NJ 08541, USA; 3 Department of Personnel Management, Work, and Organizational Psychology, Ghent University, Henri Dunantlaan 2, 9000 Ghent, Belgium

**Keywords:** cognitive abilities, specific abilities, general abilities, general mental ability, relative importance, narrow abilities, subscores, intelligence, cognitive tests

## Abstract

We review papers in the special issue regarding the great debate on general and specific abilities. Papers in the special issue either provided an empirical examination of the debate using a uniform dataset or they provided a debate commentary. Themes that run through the papers and that are discussed further here are that: (1) the importance of general and specific ability predictors will largely depend on the outcome to be predicted, (2) the effectiveness of both general and specific predictors will largely depend on the quality and breadth of how the manifest indicators are measured, and (3) research on general and specific ability predictors is alive and well and more research is warranted. We conclude by providing a review of potentially fruitful areas of future research.

## 1. Introduction

Big hammers and long nails are good for securing large items to walls and other large jobs, but they may not be useful in reupholstering a chair. Indeed, a person may be able to attach cloth over the seat of a chair with a large hammer and long nails, but large hammers may damage finished wood and exposed nails may provide unwelcome surprises for sitters. Big tools such as these are useful for their purpose, but not for every purpose. The same is true in the intelligence domain. General ability factors may be useful in predicting broad and complex outcomes, but specific abilities may be more useful determinants when the outcomes are narrower and specific to a content domain. The purpose of the special issue was to engage with the great debate regarding the usefulness of general versus specific ability predictors for an array of outcomes [1]. 

In the context of industrial and organizational (I-O) psychology (also known as work, organizational, and industrial psychology), the general versus specific abilities debate highlights a pervasive belief about the universal usefulness of the general factor, which seems to be a function of influential papers demonstrating that specific abilities contribute very little to the prediction of job or training performance after a general ability factor (itself derived from these specific factors) is accounted for (e.g., [2,3,4,5]). These papers by Ree and colleagues used large samples of military personnel, general and specific predictors derived from the Armed Services Vocational Aptitude Battery (ASVAB), and performance criteria that are collapsed across jobs (i.e., broad performance outcomes). Although one can quibble with the approach that was used by Ree and colleagues, the notion that relevant individual differences in cognitive abilities can fully be captured using a general factor has proven to be problematic for I-O psychology—particularly in the domain of selection and assessment—for a number of reasons. First, legal frameworks in some countries frequently demand that selection occurs using measures that are relevant to the job [6]. In a strict sense, a construct that only consists of a general and universal factor is not suitable for selection in the context of this legal framework [7]. Second, a general factor construct provides very limited insight into how training and development could improve performance. For instance, a company working with pilots may be interested in not only selecting highly able pilots, but also in gaining insight into how the specific limitations of individual pilots can be improved through training. Finally, an issue with a general intelligence factor is that it shows large majority–minority differences that exceed differences for most other constructs (e.g., [8]). 

The three unfavorable characteristics of intelligence as a general factor construct have effectively led to a movement in I-O psychology away from intelligence and toward other selection instruments, like assessment centers, situational judgment tests, and interviews [9]. Some of these instruments are also cognitively loaded, however, and may themselves partly measure specific intelligence factors. For example, it has been suggested that verbal and inductive abilities play a role in performance in situational interviews (e.g., [10,11]). In sum, ideas about the universal usefulness of general ability measures have stunted research on the usefulness of specific abilities for predicting work-related outcomes and the development of such measures. 

An exception to this trend is found in educational psychology and education more generally. In these fields, many practitioners and policymakers desire to provide students with feedback regarding their strengths and weaknesses in different content areas [12]. One method that many in educational disciplines believe to be a useful means for providing this diagnostic information is the reporting of content-aligned subscores in addition to overall test scores [13]. Indeed, some educational initiatives (e.g., No Child Left Behind) made the provision of diagnostic information a legal requirement, encouraging the use of subscores [14]. However, skepticism remains about the importance of what amounts to specific factors in educational measurement and psychology. The evidence that content-aligned subscores add value that is beyond the total test scores for diagnosis [13,14,15] and prediction (e.g., [16,17,18]) is equivocal, and concerns have been expressed related to the psychometric quality of these subscores [19]. 

The papers in this special issue highlight the arsenal of tools and methods intelligence researchers have at their disposal to best predict performance across contexts and general and specific criteria. Below, we review the excellent papers that were submitted as part of this special issue and provide some directions for future research. To preview the discussion, we conclude that the usefulness of the tool (i.e., general or specific abilities) depends upon the job to be done (i.e., the outcome to be predicted). 

## 2. The Special Issue

In this special issue, authors were invited to write either a) a non-empirical, theoretical, critical, or integrative review on general versus specific abilities for predicting real-world outcomes or b) an empirical analysis of a dataset to answer three questions [1]: Do the data present evidence for the usefulness of specific abilities? How important are specific abilities relative to general abilities for predicting outcomes in the dataset? Also, to what degree could/should researchers use different prediction models for the outcomes in the dataset?

Authors who chose to present an empirical paper were provided with scores on three intelligence tests from a Thurstonian test battery and school grades for German adolescents and young adults (*N* = 219). In perhaps the most straightforward empirical paper examining the contribution of general versus specific abilities for predicting school performance, Wee [20] conducted two analyses (using structural equation modeling [SEM] and a relative-importance analysis) and found that the importance of the general and specific factors depends on the criteria to be predicted. In the SEM, a general ability factor (derived from common variance among predictor ability tests) was the best predictor of a general performance factor (derived from common variance among course grades); the relative importance analysis results were also consistent with this finding. Wee [20] also found that specific abilities were the best predictor of specific course outcomes (e.g., verbal reasoning best predicted English grades in the relative importance analysis). However, the pattern of results varied across analytical approaches (e.g., verbal reasoning was not predictive of English grades in the SEM analysis after controlling for general ability and general performance). Wee attributes these differences to the diverse ways in which the factors were derived, but the difference in results—which would alter conclusions—provides an important cautionary example of how different methods can be employed to support various theoretical positions in SEM. 

Eid, Krumm, Koch, and Schulze [21] use the data that were provided to examine the contribution of general versus specific abilities on student course performance using a latent multiple regression approach that was built on bi-factor models. The description of the process for their analysis and analytic approach suggests that complex bi-factor models can result in large standard errors and difficulty in interpreting solutions (i.e., model identification and convergence problems). They go on to provide alternative approaches for examining questions about the generality of ability; that is, the extended first-order factor model and the bifactor (S-1) model. The contribution of this paper lies in its detailed description of the difficulties of applying complex models to ability data (often comprised of scores that tend to be highly correlated) and the process of trial and error that can sometimes result—even in the context of confirmatory modeling. Concrete recommendations for approaching such analyses are the fruit of their labor from which others can benefit. 

Ziegler and Peikert’s [22] approach to data analysis was similarly complex, but rather than using various methodological approaches to answer the research questions, these authors take a somewhat novel approach by assessing the changing validity of general versus specific abilities at different levels of complexity of task performance. To test their assumptions, the authors used polynomial regression and found that models containing both linear and non-linear terms outperformed the models with linear terms only—and that this effect was particularly relevant for specific (versus general) abilities. Importantly, they find that the variance that was accounted for by linear and non-linear models differed by content domain (e.g., math, German, English), suggesting that tasks in each of these domains vary in complexity and their ability demands. Unfortunately, researchers have little more than a coarse understanding of task complexity in terms of ability demands at present and this paper serves to remind us of the importance of understanding task demands that are related to criterion performance when selecting predictors. 

Although the authors of each of the three empirical papers chose to analyze the data differently, the results of all three articles point to the usefulness of both the general ability factor and specific abilities for predicting educational outcomes. Moreover, the set of papers demonstrates that the results often depend on the analytical approach adopted, a finding that should give pause to many who see modeling as an approach that unequivocally confirms a theoretical position (cf. [23]). The limitations of the individual analytical approaches notwithstanding, the empirical papers highlight the practical importance of using specific ability predictors in educational research, which is related to the design of educational interventions. Indeed, educational practitioners would likely prefer to design interventions that are focused on specific course-related material, as predicted by specific ability tests rather than relying on general ability predictors. The same might be true in the work domain, but to date we know of no research in work psychology (outside of the military context [4]) that links training needs assessment to the type of testing that is done in selection contexts. 

The authors of the remaining two papers chose a non-empirical, theoretical, critical, or integrative review to address the debate surrounding general versus specific abilities for predicting real-world outcomes. Coyle’s [24] review describes the general versus specific ability debate as the most pressing issue in intelligence research today. This review introduces new ideas regarding the meaning of the residuals that remain after general factors are partialed out of a predictor/criterion relationship (e.g., ability tilt and non-*g* residuals). Importantly, Coyle also introduces the idea that abilities will change over time through education and experience in ways that might render specific abilities increasingly important as people age (i.e., a magnification model). These ideas align well with theories of skill acquisition and cognitive aging, which highlight the importance of specific abilities (i.e., knowledge and expertise) for success in daily activities (for work and leisure) [25], and investment theories that describe skill and knowledge development as a function of the investment of attentional capacity and reasoning abilities over time [26]. Indeed, given the importance of knowledge and experience for success in daily life for older individuals, it may be that the results of intelligence research using convenience samples of college students and younger will not generalize to older populations. This is unfortunate, as the proportion of older workers continues to grow globally—particularly in industrialized countries [27], and older workers will need to be selected and trained just like younger workers are. 

Rather than call it the most important debate in intelligence research, Johnson’s [28] review describes the argument about the usefulness of general or specific ability predictors as a “tempest in a ladle” (referencing Hogan [29]). Among the salient points in this review (e.g., that pitting general and specific abilities against each other ignores their dependencies and that the importance of the general versus specific predictors will depend on the outcome), Johnson refutes Spearman’s “indifference of the indicator” stance. That is, the idea that general intelligence factors could be derived from any test or set of tests, “provided only that its correlation with *g* is equally high” ([30], p. 197). Johnson reminds readers that general factors are derived from the assessments of specific abilities that are administered (although see [31,32]). She reminds us that if specific ability assessments have certain characteristics, then the general factor will also have these characteristics, and that general factors that are divorced of content do not magically appear out of any set of specific ability assessments. On the contrary, researchers must examine the content of the manifest variables to fully understand the characteristics of the general factors that are derived from them. 

## 3. Ways Forward

In total, the papers in this special edition highlight the importance of different tools—general and specific abilities–for the prediction of an array of performance outcomes in applied settings. They also point to special considerations and cautions for the use of any tool and its accompanying analytical approach. Most salient perhaps is the idea that the value of the predictor will depend on the criterion—that predictors that are aligned with criteria in terms of breadth and content are likely to maximize prediction [33]. Below, we further expand on additional issues in the debate about the usefulness of general versus specific abilities and then describe future research directions for reinvigorating intelligence research in applied psychology.

### 3.1. Theoretical Status of Specific Abilities

One open question that becomes apparent by comparing the submissions to the special issue is the theoretical nature of specific abilities and how different models define specific abilities in different ways. For example, Wee’s [20] contribution alone included two distinct conceptualizations of the relationship between general and specific abilities, with each being aligned with a different analytic strategy. Based upon the contributions to the special issue, along with other approaches in the cognitive abilities literature (e.g., [1,2,3,4,34,35,36]), we can identify at least four distinct theoretical treatments of specific abilities: (1) Indicators of a general factor with the general factor being the source of variance for a proportion of the specific measure (i.e., *g* causes the specific abilities), (2) Orthogonal to *g*, (3) Correlated with the general factor, but without causality specification in either direction, and (4) The source of the general factor, with *g* constituting a formative composite of specific abilities or a phenomenon that emerges from the interaction of specific abilities.

Ree and colleagues’ work [2,3,4] largely takes the perspective that specific abilities are merely indicators of a general factor. By using bi-factor and relative importance approaches, however, several authors in this special issue endorsed the idea that variance that is shared by specific and general abilities does not necessarily always originate with the broader abilities. We suggest that many controversies surrounding the status of specific and general abilities may be resolved by clearly thinking through, and defining a priori, the expected relationships between general and specific abilities prior to conducting data analyses (see also [34,35,36]).

### 3.2. Indifference of the Indicator

The principle of the indifference of the indicator, summarized cavalierly in its practical aspect as “for the purpose of indicating the amount of *g* possessed by a person, any test will do just as well as any other”, is related to issues regarding the theoretical status of specific abilities ([30], p. 197). We dispute this idea and agree with Johnson’s perspective: What you put into a factor analysis largely determines what you get out, so the manifest indicators do matter. A general factor derived from the ASVAB, for example, may look very different from one that is derived from fluid reasoning tests, because individual assessments that comprise the ASVAB rely heavily on knowledge abilities [37]. 

Contrary to Spearman’s classic statement, not only will any test *not* do just as well as any other, it is often challenging to estimate *g* accurately: Using only a small number of tests often leads to poor measurement of *g*, often overestimating its importance [38]. Even when many tests are used, the test content must be sufficiently diverse to fully capture *g*’s generality and not overweight the estimate in terms of one content domain versus another [39]. When *g* estimates are extracted from large test batteries whose scores are modeled using higher-order factor analysis, those scores correlate near-unity, suggesting that they measure the same construct [31,32]. Yet, without careful attention to sample characteristics, score properties, and methodological choices, even when many tests are used to derive *g,* they do not necessarily yield identical results [40]. Adding to the complexity is the fact that, in employment testing situations, it is often not feasible to administer 10 to 20 cognitive tests to derive measures of *g*, which should make investigators cautious about interpreting their results both absolutely and when comparing the value of *g* to that of specific abilities. 

The measurement challenges for assessing *g* are only exacerbated when measuring specific abilities. By definition, specific abilities are related to narrower domains than general ability—but multiple tests are still required in order to assess specific abilities with sufficient coverage of the construct. When the number of tests is small, it is likely that researchers are confronted with a considerable level of what some have called specific factor error [41,42]. Specific factor error arises from subjects’ idiosyncratic responses to some aspect of the measurement situation (e.g., specific tests to measure a specific ability ability). For specific abilities, the accurate reliability coefficient for detecting this type of error would be a parallel test reliability coefficient between one set of tests and another independent second set of tests to measure the same specific ability. While many studies only use three or fewer indicators/tests for each specific ability, there are some notable exceptions to this rule. For example, Reeve [43] used an average of four tests as indicators of the five specific abilities in his model, Johnson and Deary [44] used an average of six tests across three specific abilities, and Jewsbury, Bowden, and Duff [45] used an average of seven tests across five specific abilities. Indeed, one of the reasons for the poor predictive performance of specific abilities relative to general ability may be that fewer indicators are used to assess them, rendering their estimates less reliable than those of *g* when a specific factor error is taken into account. Further complicating matters is that, just as the dictum of the indifference of the indicator does not always hold for general ability, it does not hold for specific abilities either. Although specific factors are often named in terms of the content of the tests that is used to define them [46], the full breadth of their influence can only be gauged using diverse content to limit specific factor error. For example, a verbal factor derived from tests that are largely composed of synonyms and antonyms will be weighted heavily toward the highly circumscribed content of those assessments; the full comprehensiveness of verbal ability would be better represented by adding sentence completions, reading passages, and vocabulary items. Accurate measurement of general ability is hard—and accurate measurement of specific abilities is even harder.

### 3.3. Different Levels of Construct Specificity and Cognitive Aging

In the ability domain, decades-old debates about the number and structure of abilities were largely settled by Carroll’s [47] reanalysis and derivation of a three-stratum structure of abilities, with *g* (GMA) at its apex, broad abilities comprising the second stratum, and narrow abilities comprising the first stratum (although see Johnson and Bouchard [48] for a competing model). The papers in this special issue have highlighted important differences between general and specific abilities, but they have not specifically addressed the second stratum of broad content abilities (e.g., fluid/reasoning abilities, crystallized/knowledge abilities). Although these broad content abilities are correlated in the population, they have different relationships with other organizationally relevant factors, such as age. Specifically, fluid ability—the ability to reason through novel problems—begins declining in late adolescence/early adulthood and continues its descent throughout the lifespan [26]. By contrast, crystallized abilities—the knowledge that is gained through experience and education—remain stable and can even increase throughout the lifespan [26]. Although age-related ability trajectories will differ across people (e.g., some 50 year olds have the ability profile of 30 year olds while others’ more resemble 70 year olds)—perhaps as a consequence of the difficulties of teasing apart knowledge versus reasoning-based strategies at the individual-level (cf. Johnson [28], Johnson & Bouchard [48]) —both longitudinal and cross sectional research demonstrate these normative patterns [49]. 

In industrialized countries, it is important for I-O practitioners and scientists engaged in testing and selection to be aware of these ability trajectories because, as mentioned earlier, most of the people who are being tested and selected are either approaching or past the age at which fluid abilities begin to decline. The median age in the United States (U.S.) labor force is currently 42 years old and increasing and similar trends can be found globally—at least in similarly industrialized countries [27]. Moreover, many so-called general ability measures are largely derived from fluid ability assessments (e.g., Raven’s Progressive Matrices and other abstract reasoning tasks) in an attempt to control for prior exposure in high-stakes assessment situations, such as selection. Because of the age-related changes in abilities described above, such measures will almost certainly put older job applicants at a disadvantage in selection. Crystallized abilities (as assessed by broad cultural knowledge measures) and general knowledge (as assessed by domain knowledge measures) are arguably more important determinants of job performance for many workers whose work engages in relatively routine tasks. However, a significant limitation in the assessment of crystallized/knowledge abilities in selection is a lack of job-relevant measures of knowledge that can be given to job applicants without prior job experience. Although some researchers have doubted that such measures would be useful [50], we encourage researchers to investigate their utility. We consider it extremely likely, for instance, that researchers can identify job-general knowledge that might transfer across many jobs (e.g., developing and managing a budget or project; motivating subordinates; writing a memo or email) that could be assessed in selection contexts in the form of assessment centers, situational judgment tests, or even paper and pencil assessments. Indeed, the need for these types of measures for selection has been highlighted by industrial and organizational psychologists [51], but much research is needed to develop and validate job general domain knowledge measures [52]. 

### 3.4. The Effect of Time on Validity Coefficients

The dynamic nature of performance over time is an additional consideration in the general versus specific ability debate that was briefly touched on by Coyle [24] and Johnson [28]. Coyle posited that general ability measures would have their highest validity for predicting early relative to later performance (e.g., [53,54]), calling into question the usefulness of such measures for selection purposes when worker tenure is long [55,56]. The reasons for these declining validities have been the subject of much debate [57,58,59], but researchers have generally converged on the idea that shifting validities are related to changes in the determinants of the criteria over time, as the task is learned [56]. 

Coyle’s [24] conclusion—that the ability determinants of performance will change as people gain expertise and skill—aligns well with theories of skill acquisition, which state that general ability is an important determinant of performance in early stages of skill acquisition when tasks require processing novel information. At later stages of skill acquisition, however, different abilities become more salient determinants of performance [60,61]. One caveat is that general ability should remain predictive of performance for inconsistent or complex skills—that is, skills that are very difficult or impossible to learn/automate. Research on skill acquisition and skilled performance also shows that the types of abilities that become more salient with skill acquisition and practice are those abilities that are more aligned with the criterion, such psychomotor ability and typing skill and verbal fluency and writing ability. Moreover, it has also been suggested that specific abilities should be key in acquiring specific types of job knowledge, while general abilities should be key in acquiring general varieties of job knowledge [62]. The consideration of time highlights the idea that both general and specific abilities may be great tools for predicting performance—but at different points in time (general ability earlier—specific abilities later). 

Most of the research that was conducted to examine the idea that different abilities will be the best predictors of performance at different stages of skill acquisition has been conducted in laboratory settings using relatively circumscribed tasks (such as skill acquisition on an air traffic control task; [60]). One exception is a longitudinal study that found that general ability was the most predictive of job performance at early stages of a job, but more specific abilities (i.e., psychomotor ability) became more predictive of job performance later, provided that job tasks were consistent. Conversely, general ability remained an important predictor over time for more complex (inconsistent) jobs [63]. With the exception of this study [63], the over-reliance on research that uses relatively short periods of time (e.g., cross sectional studies) may have biased findings in the literature systematically against detecting the effects of specific abilities. More longitudinal research is needed.

Johnson [28] also touched on the role of time when considering the relative applied value of measures of general versus specific cognitive abilities, noting that specific measures are preferable when criterion measurement takes place soon after assessment scores are gathered and vice-versa for general measures (and especially when the breadth of the criterion is matched with that of the predictor). This claim appears to contradict Coyle’s [24], but it was couched in terms that are more general than job performance, including long-term life outcomes, such as occupational attainment and longevity. Over very long periods of time, not only might the ability determinants of task performance change, so might the tasks themselves (e.g., long-tenured employees in the same organization may have very different job duties 20 years after being hired). As noted earlier, when the nature of the criterion (and its underlying constituents) is complex or obscure and the timespan for its assessment indeterminate, the broad hammer of a general ability measure may be preferable to the surgeon’s scalpel of a specific one.

### 3.5. The Criterion Problem

Above, we have made the case that the effectiveness of either general or specific ability measures for predicting performance is largely a function of what one is trying to predict (i.e., the criterion). Unfortunately, in many applied areas of research—and particularly in work psychology—the criterion is often neither well defined nor well measured [64]. We suspect that some of the debate regarding the usefulness of general versus specific abilities on the predictor side, including what we consider to be a premature conclusion that general ability is always the most effective predictor of performance, is a function of the coarseness of criterion measures. Because the criteria are relatively vague and ill defined, the use of general ability measures helps to ensure that at least some variance in the criterion will be accounted for, even though we may know relatively little about the criterion construct (e.g., whether it is uni- or multi-dimensional). To revive our earlier metaphor, if we cannot see what we are hitting, the biggest hammer is more likely than the smaller hammer to hit at least something! Similarly, the “not much more than *g*” approach [2,3,4] may be a good first swing at predicting a coarse outcome, but more precision in predictor and criterion measurement would better serve our science. 

Indeed, the multidimensional nature of performance has been known for a long time, as Toops said in 1944 “Even in simple jobs success is multidimensional” ([65], p. 274). Just because we do not measure them well, does not mean that these multidimensional facets of performance do not exist. It has been 25 years since Austin and Villanova published their seminal review of the criterion problem in I-O psychology. In that paper, they decried the lack of attention on the criterion side, particularly as compared to the intense focus on predictors (see Schmidt & Hunter [50]), among others). In the intervening two and a half decades, researchers have made small steps in recognizing two dimensions of job performance: Task performance (behavior supports the technical core) and contextual performance (behavior that contributes to the context in which work gets done [66]). Although an improvement, these dimensions continue to be relatively broad. An exception to this rule is arguably Campbell’s work on the U.S. Army’s Project A [67,68]. Campbell established relatively well defined specific job performance dimensions that were relevant to the set of jobs in the Army being studied. Re-analyses of the Project A validity data (see Kell & Lang [69] for an overview) actually supports the notion that specific abilities are related to specific criteria and they provide a window into how future work could link specific abilities to specific criteria. We hope that this special issue will serve to both revive interest in general and specific ability predictors and interest in better defining performance criteria.

## 4. Conclusions

This special issue brought a diverse group of scholars together. We thank all of the participating author teams for their excellent papers and our reviewers for their insight and helpful and constructive comments. In our initial call for this special issue, we provided a typical educational dataset and asked potential contributors three questions: Do the data present evidence for the usefulness of specific abilities? How important are specific abilities relative to general abilities for predicting outcomes in the dataset? Also, to what degree could/should researchers use different prediction models for the outcomes in the dataset? Our hope in starting with a typical dataset was to gain diverse and new insights beyond the general notion that there is “not much more than *g*” when it comes to linking intelligence to outcome criteria. Most researchers and practitioners working with intelligence measures face similar questions and datasets. As we noted in the introduction to this comment, there has long been a notion in the intelligence literature that the answer to all three questions is clearly: No, not important, and different prediction models are unnecessary. As we suggested above, the focus on *g* in the applied intelligence literature has potentially long hampered progress and innovation in the field. The three empirical papers and two commentaries provide a set of novel perspectives and ideas that are new to us. The contributions show that research on general and specific abilities is alive and well, and describe how a focus on specific abilities can help researchers and practitioners gain valuable additional insights into the determinants of performance over general abilities. The contributions also demonstrate how researchers can simultaneously consider general and specific measures in their research and balance and reconcile the opposing viewpoints on their potential benefits. We believe that these ideas provide a building block for more balanced and informed perspectives on the role of general and specific abilities and future progress in applied research on intelligence.
